# Supplementary material for: In vivo cyclic overexpression of Yamanaka factors restricted to neurons reverses age-associated phenotypes and enhances memory performance
Source: Commun Biol. 2024 May 24;7:631. doi: 10.1038/s42003-024-06328-w (PMC11126596; doi:10.1038/s42003-024-06328-w)
Supplement: Supplementary file 2 — Supplementary information [file 42003_2024_6328_MOESM2_ESM.pdf]

In vivo cyclic overexpression of Yamanaka factors restricted to neurons reverses age-associated phenotypes and enhances memory performance

Alejandro Antón-Fernández [1,2 *#], Marta Roldán-Lázaro[1*], Laura Vallés-Saiz[1], Jesús Ávila[1,2], Félix Hernández [1,3#]

[1] Centro de Biología Molecular "Severo Ochoa" (UAM-CSIC). Nicolás Cabrera, 1. Cantoblanco. 28049 Madrid, Spain

[2] Consejo Superior de Investigaciones Científicas (CSIC). Serrano 117. 28006 Madrid, Spain

[3] Lead Contact

*Equal contribution

#Correspondence: fhernandez@cbm.csic.es (FH) and aanton@cbm.csic.es (AAF)

Inventory of Supplementary Figures

1. Supplementary figure 1. Transcriptomic and histological characterization of α-CaMKII-OSKM young mice with continuous induction from birth.

2. Supplementary figure 2. Protein expression of the Yamanaka factor Klf4 at various time points during a cyclical induction protocol.

3. Supplementary figure 3. Yamanaka factors expression during continuous neuronal partial reprogramming in adult mice.

4. Supplementary figure 4. Correlation between Klf4 protein expression and C-fos+ cells.

5. Supplementary figure 5. Immunofluorescence results for Aggrecan in continuous neuronal partial reprogramming from birth to 3 months old mice.

6. Supplementary figure 6. Detection of Aggrecan protein expression by Western Blot.

7. Supplementary figure 7. Immunofluorescence results for the analysis of neurogenesis in the dentate gyrus in cyclic neuronal partial reprogramming in adult mice.

Supplementary Figures

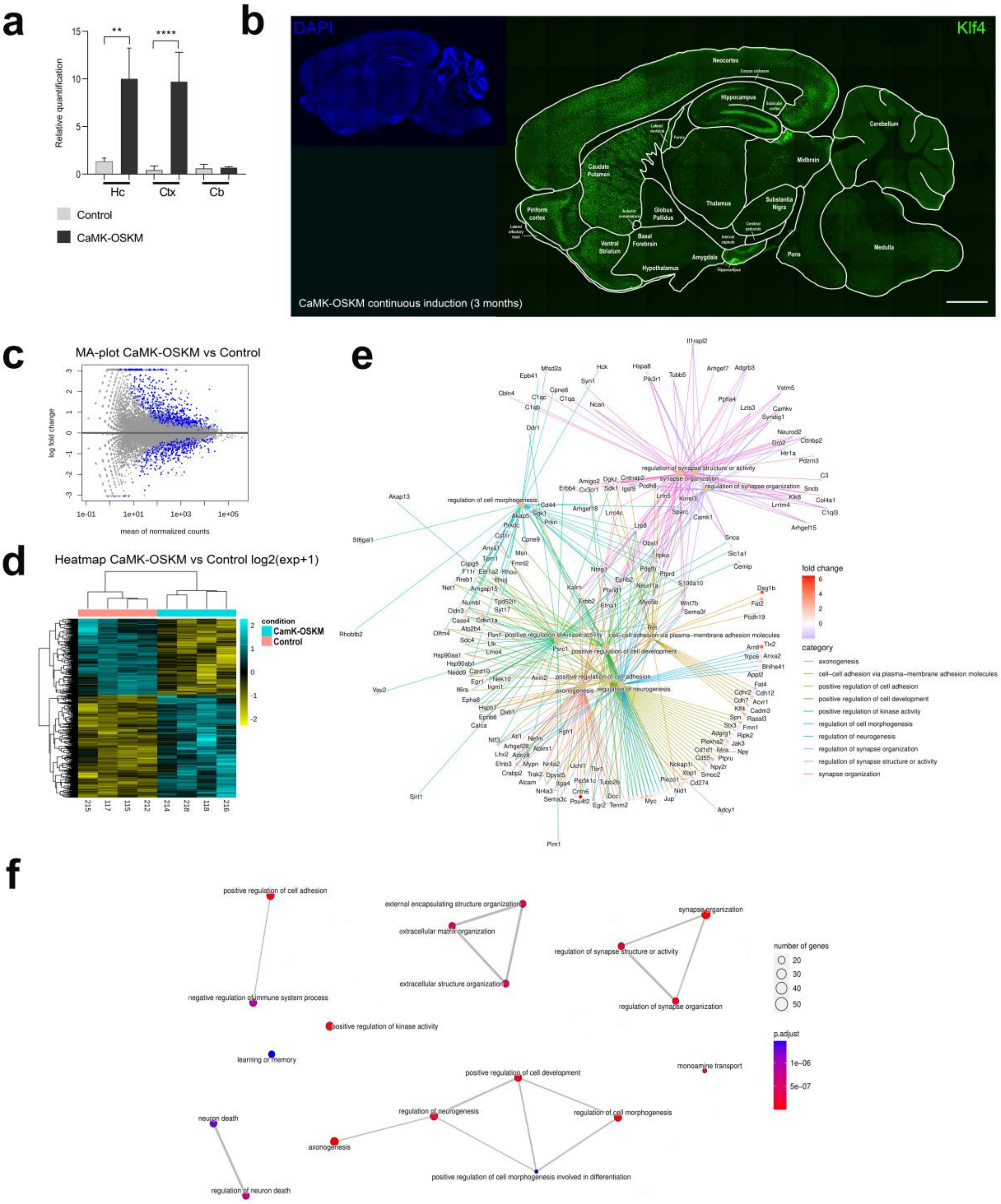

Supplementary figure 1. Transcriptomic and histological characterization of α-CaMKII-OSKM young mice with continuous induction from birth.

(a) Graphical representation of the mean ± SEM of the relative quantification obtained from quantitative RT-PCR of the transgene OSKM in hippocampus, neocortex and cerebellum (control n=10, α-CaMKII-OSKM mice n=5). Data were normalised to GAPDH. $*p < 0.05$, $** p < 0.01$, $*** p < 0.001$, $**** p < 0.0001$

(b) Representative microphotographs of the immunoreactivity obtained for Klf4 in the brain of a 3 months old mouse with continuous induction from birth. Scale bar shown indicates 1000 μm. Approximate representation of the different brain areas are shown.

(c) MA-plot which represents genes coloured in blue that have q-values less than 0.05. Points which fall out of the window are plotted as open triangles pointing either up or down.

(d) Heatmap where the data are displayed in a grid where each row corresponds to a gene and each column to a sample (from two different conditions). The colour and intensity in the heatmap represent changes of gene expression.

(e) Cnetplot from hippocampal samples, showing Gene-Concept Network from GO ORA data (Gene Ontology over-representation analysis). The data show several functionally related gene groups, whose transcription has been significantly altered during the expression of Yamanaka factors in cortical neurons.

(f) Emapplot from hippocampal samples, showing an Enrichment Map for enrichment results of over-representation test or gene set enrichment analysis. The data demonstrate changes related to reprogramming processes that are also affecting the transcription of neuron-specific genes such as those associated with synapse organisation and formation.

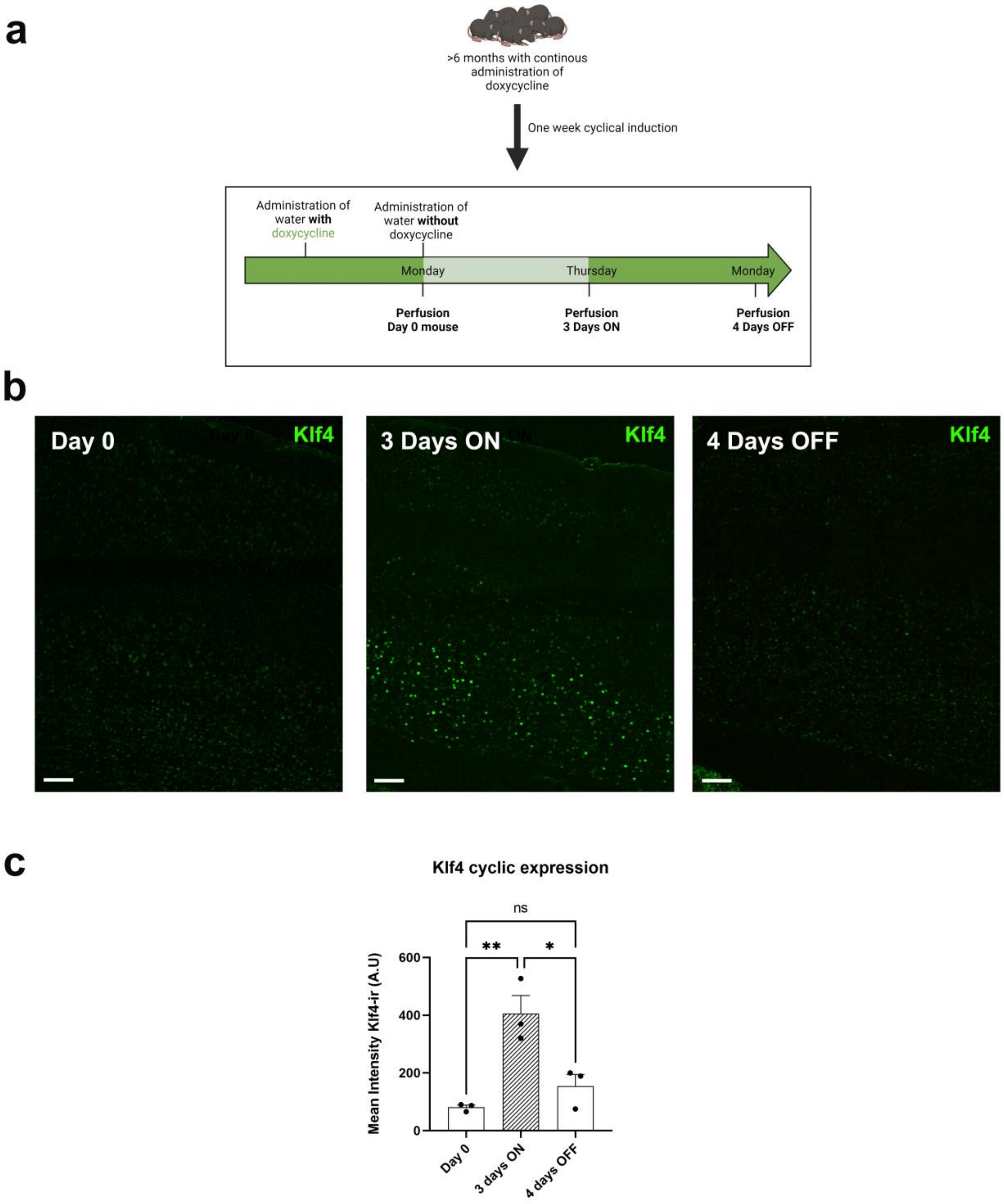

Supplementary figure 2. Protein expression of the Yamanaka factor Klf4 at various time points during a cyclical induction protocol.

(a) Scheme of the protocol used for the experiment, showing the timeline of the different transgene induction intervals at which the various mice from the same litter were perfused. After 6 months of consuming doxycycline in drinking water, one litter underwent a single week of cyclic induction. Double transgenic α-CaMKII-OSKM mice were perfused prior to the start of the induction (day 0), which in the protocol we followed corresponded to a Monday. On Thursday, after 3 days of induction (water without doxycycline), α-CaMKII-OSKM mice were perfused, and the following Monday, before the new induction cycle began, the remaining double transgenic mice were perfused.
(b) Representative microphotographs of immunoreactivity obtained for Klf4 in the somatosensory neocortex from α-CaMKII-OSKM mice perfused at different days of the week during cyclical induction protocol. Scale bar shown indicates 100 µm.
(c) Graphical representation of the mean ± SEM of the mean intensity (arbitrary units) of Klf4 immunoreactivity in the somatosensory neocortex of α-CaMKII-OSKM mice. One-way ANOVA was conducted ($F = 15.65$; p-value < 0.0042), revealing significant differences after the 3-day induction

protocol compared to day 0 (p-value=0.0043) and after 4 days of induction inhibition with doxycycline administration (p-value= 0.0146). **p<0.01, *p < 0.05 by Tukey multiple comparisons tests.

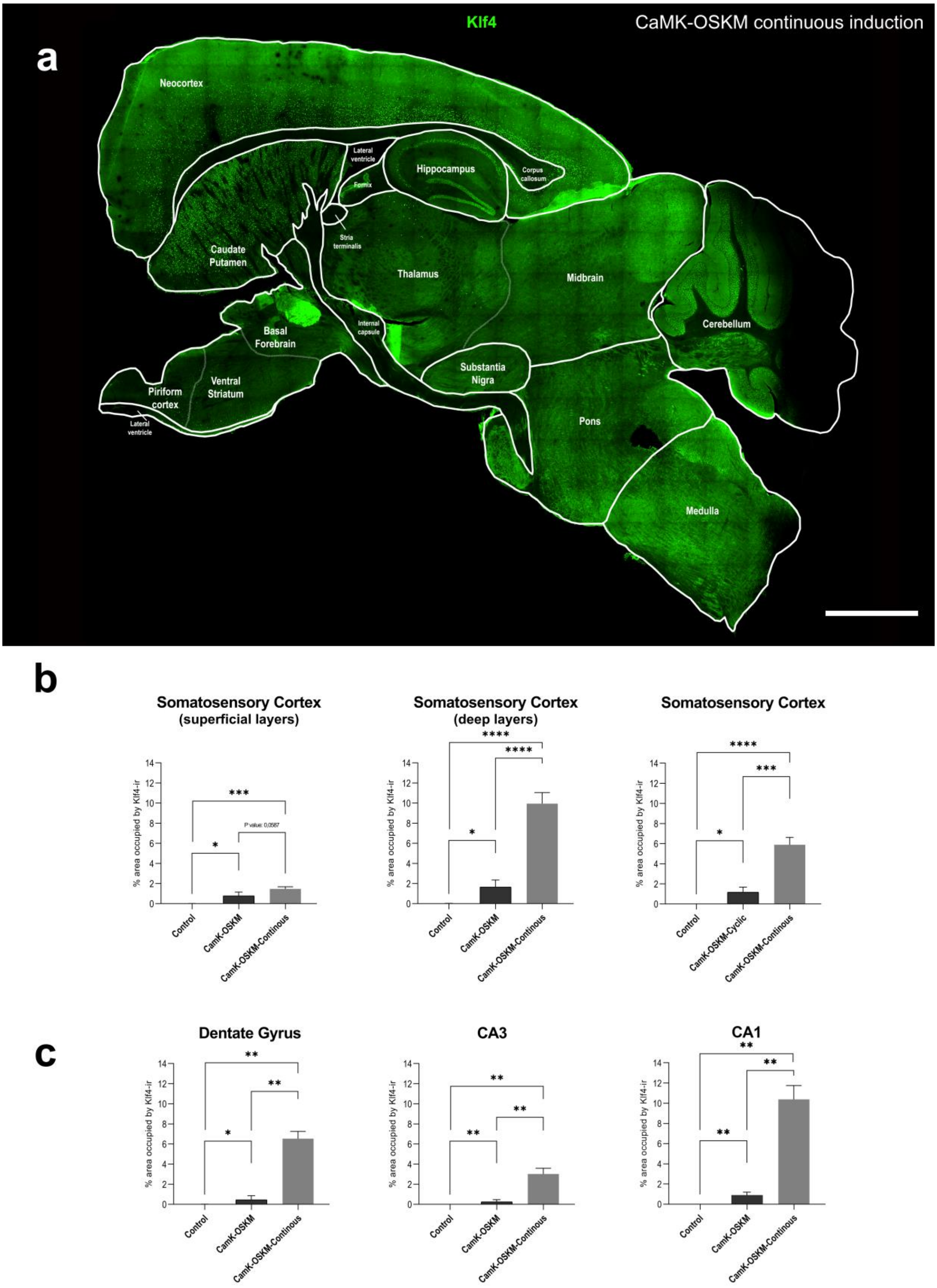

Supplementary figure 3. Yamanaka factors expression during continuous neuronal partial reprogramming in adult mice.

(a) Representative microphotographs of Klf4 Yamanaka Factor immunoreactivity (in green) obtained from sagittal brain sections of α-CaMKII-OSKM mice with continuous transgene induction from 6 to 11 months of age. Schematic outlines approximating the boundaries of some of the central nervous

system's most relevant areas have been overlaid on the microphotographs. Scale bar shown in A indicates 1000 μm.

(b,c) Graphical representation of the mean ± SEM of the percentage of occupied area by Klf4-immunoreactivity in somatosensory neocortex, distinguishing deep layers from superficial (b) and different regions from hippocampal region, including CA1, CA3 and dentate gyrus (c). One-way Anova was performed between control 11 months old adult mice (OSKM mice without α-CamKII-tTA transactivator; n = 5), α-CaMKII-OSKM mice with cyclic induction (n = 9) and with continuous induction (n = 7). Results showed differences between different groups for data from somatosensory cortex (F = 29.59; p-value < 0.0001), distinguishing superficial (F = 9.235; p-value = 0.0028) and deep layers (F = 40.36; p-value < 0.0001). Kruskal-Wallis was performed for different regions of the hippocampus, dentate gyrus (Kruskal-Wallis statistic (KWs) = 12.80; p-value < 0.0001), CA3 (KWs = 13.83; p-value < 0.0001) and CA1 (KWs = 14.29; p-value < 0.0001), reporting significant differences regarding KLF4 YF expression between different groups. Continuous transgene induction in α-CaMKII-OSKM mice led to higher expression of KLF4 protein in comparison with cyclical induction and with control transgenic mice. Cyclical induction led also to higher expression of KLF4 protein expression regarding control transgenic mice. *p < 0.05, ** p < 0.01, *** p < 0.001, **** p < 0.0001 by Student’s paired t-test.

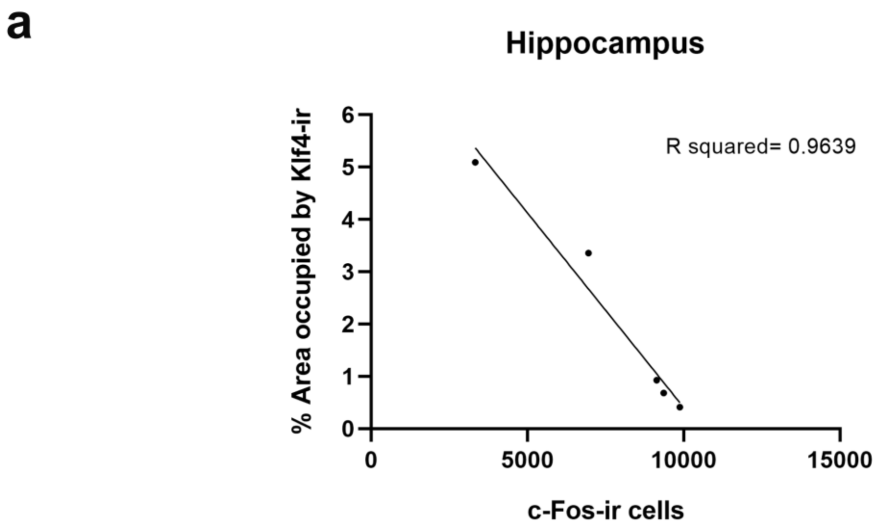

Supplementary figure 4. Correlation between Klf4 protein expression and C-fos+ cells in cyclic neuronal partial reprogramming in adult mice.

Pearson's correlation and simple linear regression between Klf4-ir expression (% area occupied) and respective C-fos+ cell densities in total hippocampus. Both data were obtained from the same hippocampus of α-CaMKII-OSKM mice. R^2: Pearson's correlation coefficient. (p-value=0.0029).

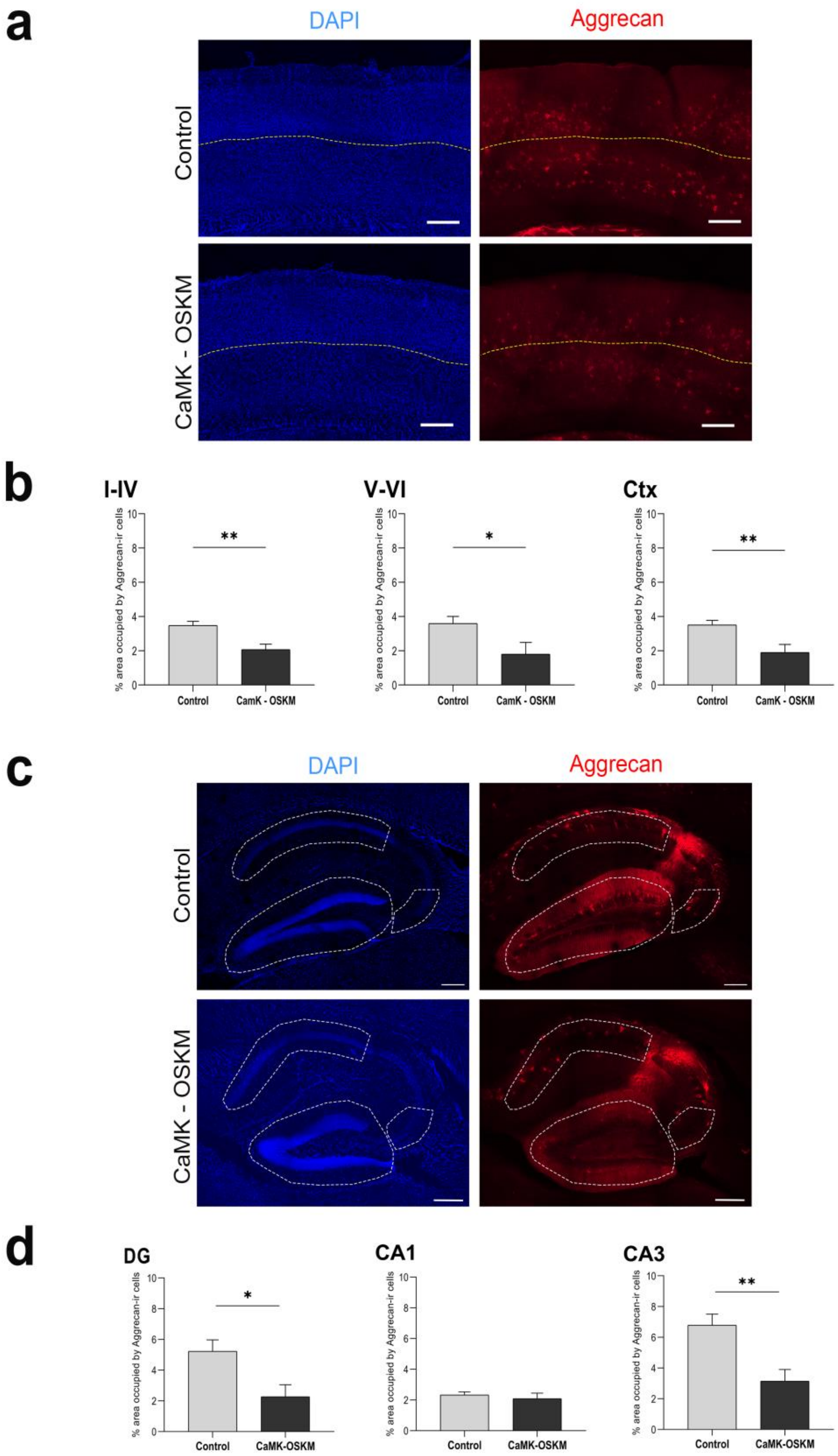

Supplementary figure 5. Immunofluorescence results for Aggrecan in continuous neuronal partial reprogramming from birth to 3 months old mice.

(a) Representative microphotographs of immunoreactivity obtained for Aggrecan in the neocortex from control (n = 11) and α-CaMKII-OSKM mice (n = 4). Scale bar shown indicates 200 µm.

(b) Graphical representation of the mean ± SEM of the percentage of area occupied by Aggrecan-immunoreactive cells per mm3 in the neocortex. *$p < 0.05$, ** $p < 0.01$. A generalised decrease is

observed throughout the somatosensory cortex (p-value = 0.0049), occurring in both upper layers (p-value = 0.0037) and deep layers (p-value = 0.0200).
(c) Representative microphotographs of immunoreactivity obtained for Aggrecan in the hippocampus of the different murine models. Scale bar shown indicates 200 μm.
(d) Representation of the mean ± SEM of the percentage of area occupied by Aggrecan immunoreactive per mm3 in the hippocampus. *p < 0.05, ** p < 0.01. Likewise, a decrease in the hippocampal formation is observed, being more marked in CA3 (p-value = 0.0058) and in the dentate gyrus (p-value = 0.0180). However, no significant differences were observed in CA1.

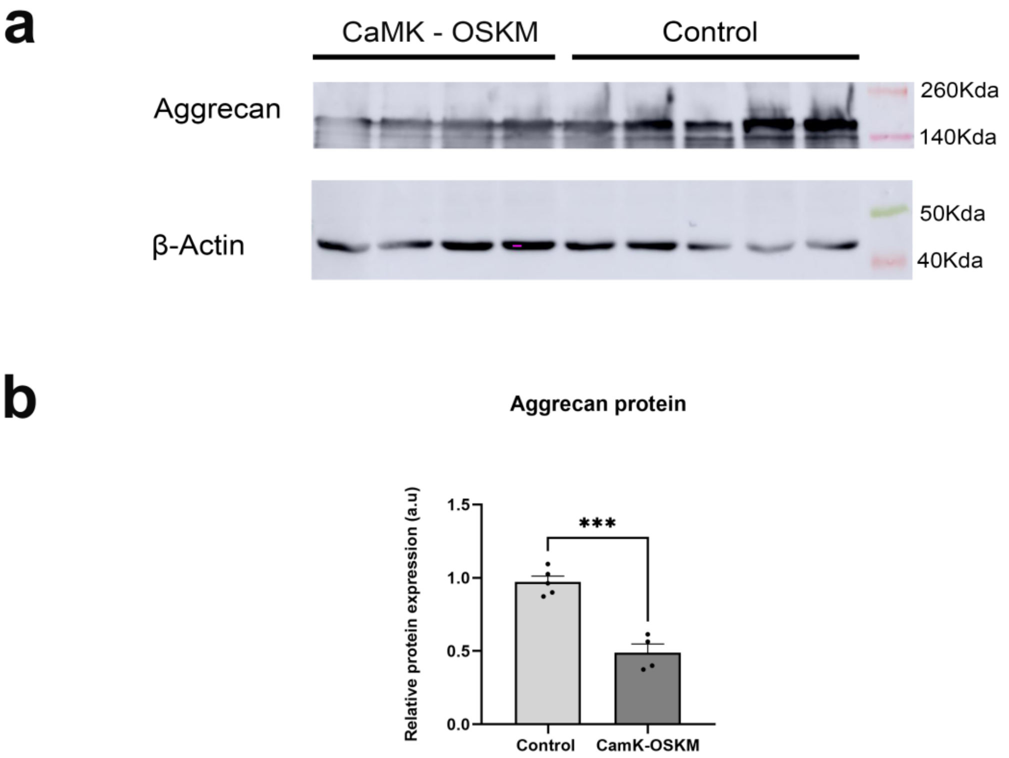

Supplementary figure 6. Detection of Aggrecan protein expression by Western Blot.
(a) Representative examples of Western blot of neocortical brain homogenates from CamK-OSKM and control mice using the polyclonal aggrecan antibody and β-actin as loading control. (b) Quantification of aggrecan protein. The intensity of the signal from Aggrecan immunoreactivity was quantified relative to the β-actin signal detected in the same animal. Data are presented as mean ± SD from three independent replicates. (*** p < 0.001).

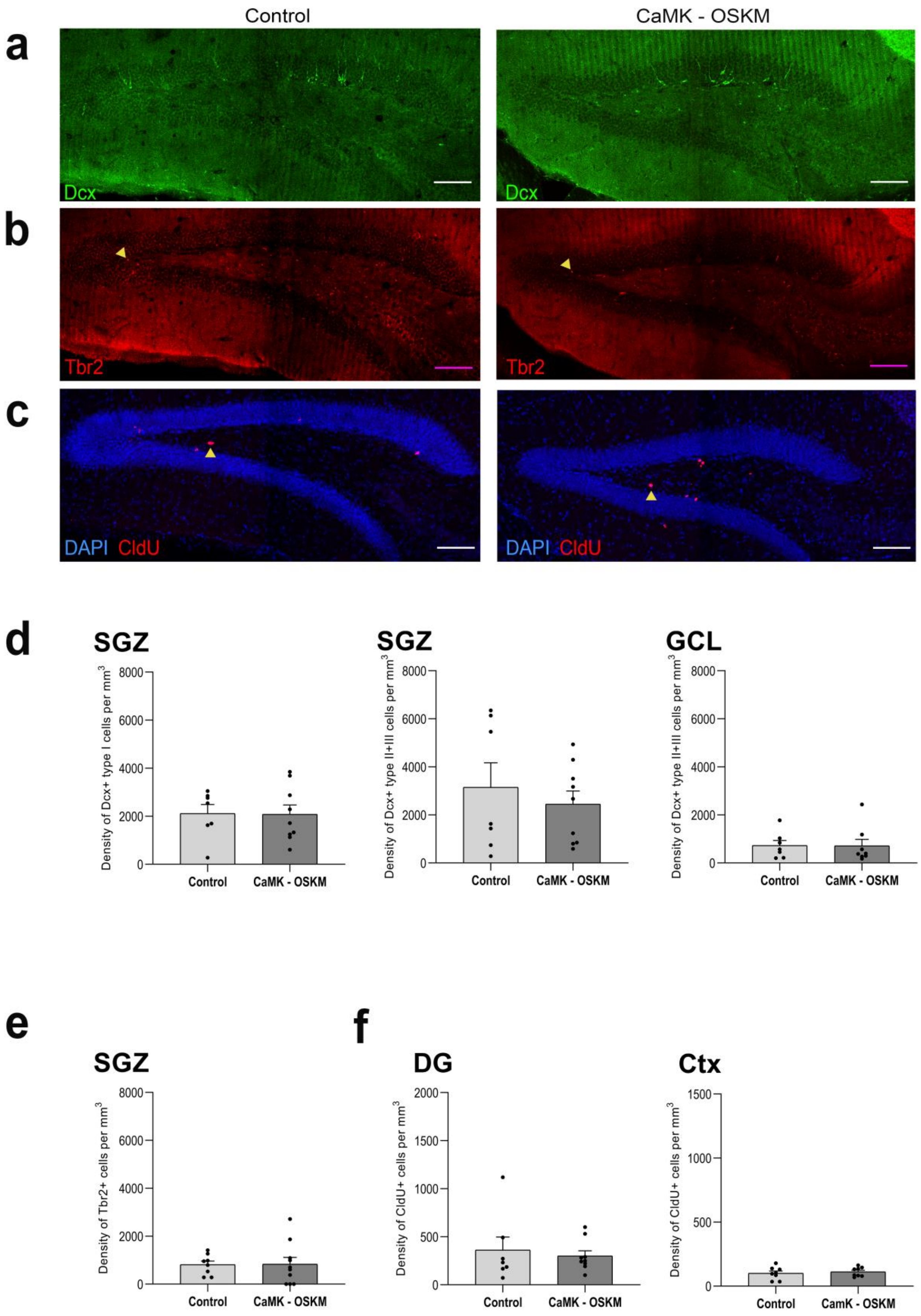

Supplementary figure 7. Immunofluorescence results for the analysis of neurogenesis in the dentate gyrus in cyclic neuronal partial reprogramming in adult mice.

(a-c) Representative microphotographs of immunoreactivity obtained for Doublecortin (Dcx) (a), Tbr2 (b) and CldU (c) in the neocortex from control (left side) and α-CaMKII-OSKM mice (right side). Yellow arrowheads are shown in b and c, indicating Tbr2 and CldU-immunoreactive cells, respectively. Scale bar shown indicates 100 μm.

(d) Graphical representation of the mean ± SEM of the density of Dcx-positive cells per mm3 differentiating between neuronal types I and II-III studied in subgranular zone and granular cell layer. No significant differences were observed between the experimental groups, control (n = 7) and α-CaMKII-OSKM mice (n = 9).

(e) Graphical representation of the mean ± SEM of the density of Tbr2-positive cells per mm3 in the subgranular zone. No significant differences were observed between the experimental groups, control (n = 8) and α-CaMKII-OSKM mice (n = 10).

(f) Graphical representation of the mean ± SEM of the density of CldU-positive cells per mm3 in the dentate gyrus (subgranular zone and granular cell layer). No significant differences were observed between the experimental groups, control (n = 8) and α-CaMKII-OSKM mice (n = 10).
