# Supplementary material for: In vivo cyclic overexpression of Yamanaka factors restricted to neurons reverses age-associated phenotypes and enhances memory performance
Source: Commun Biol. 2024 May 24;7:631. doi: 10.1038/s42003-024-06328-w (PMC11126596; doi:10.1038/s42003-024-06328-w)
Supplement: Supplementary file 3 — Description of additional supplementary files [file 42003_2024_6328_MOESM3_ESM.docx]

Description of Additional Supplementary Files

**File name:** Supplementary Data 1

**Description:** Differentially expressed genes from transgenic animals that were not administered doxycycline from birth.

**File name:** Supplementary Data 2

**Description:** Transcriptomic data from transgenic animals with cyclic expression of Yamanaka Factors in adult age.
